# The Role of Cytoreductive Surgery Plus Hyperthermic Intraperitoneal Chemotherapy (HIPEC) in Peritoneal Metastases from Breast Cancer: A Comprehensive Review and Pooled Individual-Patient Analysis

**DOI:** 10.3390/jcm15124511

**Published:** 2026-06-11

**Authors:** Dimitrios Papageorgiou, Vasileios Kalles, Vasilios Pergialiotis, Ioannis K. Papapanagiotou, Nikolaos Tasis, Savvas Petrogiannis, Katerina Papakonstantinou, Ioakeim Sapantzoglou

**Affiliations:** 1 Department of Gynecology, Athens Naval and Veterans Hospital, 11521 Athens, Greece; 2 Department of Surgery, Athens Naval and Veterans Hospital, 11521 Athens, Greece; 3 1st Department of Obstetrics and Gynecology, National and Kapodistrian University of Athens, 10679 Athens, Greece; 4 Laboratory of Anatomy, Faculty of Nursing, National and Kapodistrian University of Athens, 11527 Athens, Greece

**Keywords:** breast cancer, peritoneal metastases, cytoreductive surgery, cytoreduction, HIPEC, peritoneal carcinomatosis, metastatic breast cancer

## Abstract

**Background/Objectives**: Peritoneal metastases from breast cancer (PMBC) are rare, aggressive, and lack standardized management. Cytoreductive surgery (CRS) combined with hyperthermic intraperitoneal chemotherapy (HIPEC) has emerged as a potential locoregional strategy for highly selected patients. This PRISMA-informed narrative review used a structured and reproducible search and study-selection process, SWiM-guided narrative synthesis, and descriptive pooled individual-patient data (IPD) analysis to evaluate the feasibility, safety, and reported oncologic outcomes of CRS plus HIPEC in PMBC. **Methods**: The English-language literature was searched in PubMed/MEDLINE, Embase, and Google Scholar up to 31 December 2025. Eligible peer-reviewed full-text articles reported PMBC patients treated with CRS plus HIPEC and at least one perioperative or oncologic outcome. Patient-level data were extracted when explicitly reported and were summarized descriptively; no inferential survival analysis was performed. Risk of bias was assessed using JBI checklists for case reports/series and ROBINS-I for the multicenter cohort. **Results**: Six peer-reviewed studies were included (66 PMBC patients); 30 patients underwent CRS plus HIPEC. Five studies provided sufficient patient-level data for descriptive pooled IPD analysis (*n* = 17). Median age at CRS/HIPEC was 56 years (*n* = 13 with reported age), and the median interval between breast cancer diagnosis and PMBC was 12 years (range 0–30 years; available-case analysis). Median PCI was 21.5 (*n* = 16), and complete cytoreduction (CC-0) was achieved in 9 of 17 patients. Major postoperative morbidity occurred in 17.6%, while no in-hospital or 30-day mortality was reported. Reported disease-control and survival outcomes were heterogeneous and are therefore summarized only descriptively. In the multicenter cohort, curative-intent CRS with or without HIPEC was associated with a median overall survival of 61.5 months measured from diagnosis of peritoneal metastases; however, HIPEC-specific baseline characteristics and oncologic outcomes were not separately stratified. **Conclusions**: CRS plus HIPEC appears feasible in highly selected PMBC patients and may be associated with favorable outcomes when complete cytoreduction is achievable. However, the evidence is sparse, heterogeneous, and highly prone to selection and publication bias. Therefore, no causal inference regarding the independent benefit of HIPEC can be drawn, and this approach should be considered investigational pending prospective multicenter registries.

## 1. Introduction

Although breast cancer survival rates are among the highest of all cancer types [1], metastatic breast cancer remains a challenging clinical scenario with poor long-term outcomes [2,3]. Peritoneal metastases from breast cancer (PMBC) represent an uncommon and clinically challenging pattern of spread. Available retrospective series of peritoneal disease from breast cancer managed with systemic therapy and/or palliative interventions have reported very poor outcomes; for example, Tuthill et al. described a median survival of 1.5 months in a heavily symptomatic cohort, highlighting the need for focused clinical research in this population [4]. These estimates likely reflect selected palliative populations and should be interpreted in context of disease burden, histology, and treatment era [3,4].

Recently, in an effort to integrate local and regional treatments, the combination of surgical cytoreduction with the administration of intraperitoneal chemotherapy has emerged as a novel method to resect all visible disease as well as treat microscopic residual disease [5]. Cytoreductive surgery (CRS) demands the removal of considerable areas of visceral organs in order to achieve optimal tumor reduction status. Towards this end and with the aim of achieving complete cytoreduction (CC-0), various peritoneal excision techniques have been described [6,7,8]. This approach has now been adopted widely in malignancies such as pseudomyxoma peritonei from appendiceal cancer and has been shown to have encouraging results in the treatment of peritoneal metastases from ovarian and colorectal cancer [9,10,11]. These advanced surgical techniques utilized to improve oncologic outcomes may result in significant consequences, such as surgical menopause, in female patients of reproductive age. Despite this risk, cases of successful pregnancies after CRS and HIPEC have been published, suggesting that the increased survival after such procedures offers hope for fertility preservation and childbearing in selected patients [12].

In the case of breast cancer, systemic chemotherapy has been the mainstay for breast cancer peritoneal metastatic disease, with surgery being indicated for palliative purposes only [13]. However, as the experience of surgeons and oncologists in cytoreductive surgery and intraperitoneal chemotherapy has been rapidly growing, there is emerging research on the use of this approach in “unusual” cases such as breast cancer patients [6]. Therefore, the aim of this study is to comprehensively synthesize the published clinical evidence on CRS with HIPEC for PMBC, focusing on patient selection and disease characteristics, feasibility and perioperative safety, and oncologic outcomes including intraperitoneal disease control and survival. By clearly distinguishing between what current data support and what remains uncertain, we also seek to identify priorities for future prospective registries and multicenter studies.

## 2. Materials and Methods

This article was designed as a narrative review with a structured, reproducible literature search and transparent study-selection reporting. PRISMA 2020 items [14] were used to report the identification, screening, eligibility, and inclusion of records, while synthesis was performed narratively according to SWiM principles where applicable [15]. The available evidence consists predominantly of case reports, small case series, and one non-randomized multicenter cohort; therefore, no effect-size meta-analysis was planned and this work was not designed as a formal systematic review. Accordingly, the protocol was not registered in PROSPERO. Before study selection, the authors defined the research question, eligibility criteria, outcomes of interest, and synthesis strategy.

### 2.1. Search Strategy

Literature searches were conducted in PubMed/MEDLINE, Embase, and Google Scholar up to 31 December 2025. The search strategy combined three concept blocks: breast cancer terms, HIPEC/intraperitoneal hyperthermic chemotherapy terms, and peritoneal metastasis/peritoneal carcinomatosis terms. Database-specific search strings, field tags, date limits, and PRISMA counts are provided in Supplementary Section S3. Database-specific yields were PubMed/MEDLINE (*n* = 18) and Embase (*n* = 57). Google Scholar was screened using equivalent keyword combinations, and the first 1163 relevance-ranked results were reviewed. Reference lists of eligible reports were also manually checked. Duplicate records were removed using DOI matching and exact or near-exact title matching, with author/year verification when needed.

### 2.2. Eligibility Criteria

Eligible studies were peer-reviewed full-text articles in English reporting patients with peritoneal carcinomatosis from breast cancer treated with CRS combined with HIPEC. All clinical study designs were eligible for inclusion. Studies were required to report at least one of the following: perioperative outcomes or oncologic outcomes. Exclusion criteria were review articles, editorials, conference abstracts without an accompanying full text, book chapters, non-human studies and reports in which peritoneal disease was not attributable to a breast primary.

### 2.3. Study Selection

Titles and abstracts of articles retrieved by the initial search were independently screened by two authors, to determine those articles for full-text review. Any discrepancies concerning the evaluation of the studies were arbitrated by all authors. Moreover, the reference lists of all eligible studies were assessed for additional articles. Reasons for exclusion at the full-text stage were recorded.

### 2.4. Data Extraction

For each patient, data were extracted about their demographic characteristics, primary breast cancer characteristics, initial surgical and adjuvant management, interval between primary malignancy and peritoneal metastasis, peritoneal metastasis characteristics, technical aspects of CRS and HIPEC (peritoneal carcinomatosis index—PCI; completeness of cytoreduction—CC; medications, duration and temperature during HIPEC; blood loss; intensive care unit (ICU) length of stay; postoperative length of stay and morbidity/mortality), and oncological outcomes (progression-free and overall survival).

### 2.5. Risk-of-Bias Assessment

Given that the evidence base comprises predominantly uncontrolled case re-ports/case series and one non-randomized observational cohort, we applied design-appropriate appraisal tools rather than a single generic scale. For case reports and case series we used the Joanna Briggs Institute (JBI) critical appraisal checklists (case report: 8 items, case series: 10 items) [16], which evaluate reporting completeness and key sources of bias such as clarity of inclusion criteria, standardized measurement of disease and outcomes and completeness/consecutiveness of case inclusion. For the multicenter observational cohort, which presents an intent-of-treatment comparison, we used the Risk Of Bias In Non-randomized Studies of Interventions (ROBINS-I) framework [17] to assess bias due to confounding, selection of participants, classification of interventions, deviations from intended interventions, missing data, outcome measurement and selective reporting. Two reviewers independently completed the checklists and disagreements were resolved by consensus. We did not calculate a numeric quality score. Instead, we summarized study-level judgments qualitatively (low/moderate/high concerns for JBI-based appraisals and low/moderate/serious/critical risk for ROBINS-I) and used these judgments to guide a conservative, primarily descriptive synthesis.

### 2.6. Data Synthesis and Statistical Analysis

Given the rarity of PMBC treated with CRS plus HIPEC and the heterogeneity of study designs, patient selection, reporting, and outcome definitions, the primary synthesis was a structured narrative synthesis guided by SWiM principles [15]. Where patient-level data were explicitly available in case reports or case series, an IPD dataset was constructed and is summarized descriptively using available-case denominators. Continuous variables are summarized as medians, ranges, means, or interquartile ranges according to the level of reporting in the source articles; categorical variables are summarized as counts and proportions. No comparative effect-size meta-analysis, hypothesis testing, or model-based survival curve was performed. Disease-control and survival outcomes are reported only descriptively as presented in the source articles. The multicenter survey, which reports outcomes for curative-intent CRS with or without HIPEC versus non-curative strategies, was synthesized separately at the cohort level to avoid double counting and to provide context on patient selection and comparative intent-of-treatment outcomes.

## 3. Results

### 3.1. Study Characteristics

**Figure 1:**
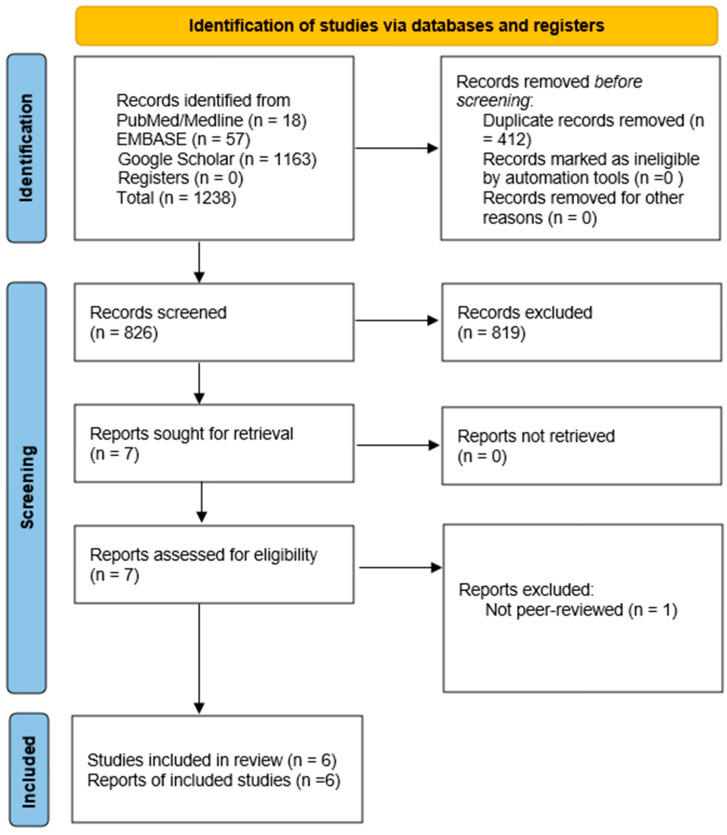
PRISMA 2020 flow diagram.

A total of 1238 records were identified (PubMed/MEDLINE *n* = 18; Embase *n* = 57; Google Scholar screened *n* = 1163). After removal of 412 duplicates, 826 records were screened and 819 were excluded at title/abstract screening. Seven full-text reports were assessed for eligibility; one was excluded as non-peer-reviewed (book chapter), and six peer-reviewed studies were included (Figure 1). Overall, the included studies enlisted 66 patients with peritoneal metastases from breast cancer. Out of 66 patients, 30 were treated with CRS combined with HIPEC.

Among the six included studies, five (case reports and small case series) provided sufficient details for extraction of individual-patient data, producing a pooled dataset of 17 patients treated with CRS combined with HIPEC [18,19,20,21,22]. The remaining study is a multicenter retrospective cohort of 49 patients, evaluating curative-intent CRS with or without HIPEC (20 patients) compared with non-curative strategies (29 patients) [23], and was therefore analyzed separately at the cohort level to avoid double counting and methodological overlap. Study-level characteristics are summarized in Table 1.

### 3.2. Individual-Patient Dataset

#### 3.2.1. Patient Characteristics

Across 17 patients included in the IPD dataset [19,20,21,22,23], the median age at CRS/HIPEC was 56 years (range, 45–77 years; *n* = 13 with reported age). The interval between primary breast cancer diagnosis and diagnosis of peritoneal metastases varied widely across reports and included synchronous presentations; using available-case patient-level data (*n* = 15), the median interval was 12 years (IQR 8–15; range 0–30 years) (Table 2).

#### 3.2.2. CRS + HIPEC Characteristics

At the time of CRS and HIPEC, most patients had disease confined to the peritoneal cavity or minimal extra-peritoneal involvement. The median PCI score was 21.5 (range, 7–39; *n* = 16), and complete cytoreduction (CC-0) was achieved in 9 of 17 patients (Table 2). Across studies reporting these perioperative metrics, the mean operative time was 374.5 min (range, 190–636; *n* = 11), the mean HIPEC duration was 65.3 min (range, 60–90; *n* = 17), and reported perfusion temperatures ranged from 40 °C to 43 °C (Table 3).

#### 3.2.3. Postoperative Course

Across studies reporting postoperative course, the mean intensive care unit (ICU) stay was 18.9 h (range, 12–24; *n* = 7) and the mean postoperative hospital stay was 15.1 days (range, 8–24; *n* = 12) (Table 3). Major postoperative complications (Clavien–Dindo ≥ III or CTCAE ≥ grade 3, as reported in the primary studies) occurred in 3 of 17 patients (17.6%), while HIPEC-related toxicities were predominantly hematologic and reversible. No in-hospital or 30-day mortality was reported.

Disease-control and survival outcomes were reported heterogeneously across case-based studies and are presented descriptively only. Across the pooled IPD dataset, reported follow-up or survival durations ranged from 10 to 128 months, and four deaths were reported during follow-up. Disease-free survival and progression-free survival were inconsistently defined across reports, with explicit disease-free durations ranging from 13 to 128 months and progression-free survival ranging from 2 to 5 months in the two cases reported by Barakat et al. Because the pooled dataset included only 17 highly selected patients from case reports and small case series, no model-based survival curve or time-point survival estimates were generated.

#### 3.2.4. Histology Data

Histology was reported as invasive ductal carcinoma in 8 of 17 cases, invasive lobular carcinoma in 7 of 17 cases, and mixed ductal–lobular carcinoma in 1 case (1 case not reported). Thus, lobular histology accounted for 7/16 (43.8%) among patients with reported histology. In the multicenter cohort (curative-intent group), lobular histology was also frequent (13/20; 65%) (Table 1). Receptor status and molecular subtype were inconsistently reported across case-based studies; where available, most cases were hormone-receptor-positive (Table 2). These observations are exploratory and intended to inform hypothesis generation rather than confirm histology- or subtype-specific effects.

### 3.3. Multicenter Cohort

In the multicenter cohort by Cardi et al. [23], 49 patients with peritoneal metastases from breast cancer were included. Of these, 20 patients were selected by a multidisciplinary team for curative-intent surgery, consisting of CRS with or without HIPEC, while 29 patients received non-curative treatments such as palliative surgery, neoadjuvant intraperitoneal chemotherapy (NIPEC), laparoscopic HIPEC for malignant ascites, or pressurized intraperitoneal aerosol chemotherapy (PIPAC). Within the curative-intent cohort, CRS was performed in all 20 patients, while HIPEC was administered in 13 patients, according to institutional protocols and intraoperative assessment.

The authors did not report baseline characteristics separately for these 13 HIPEC-treated patients. Specifically, age, interval from primary breast cancer diagnosis, histology, molecular subtype, PCI score and completeness of cytoreduction were not stratified by HIPEC use. Accordingly, these variables are presented only for the overall curative-intent cohort (CRS ± HIPEC, *n* = 20) and are not attributed to the CRS + HIPEC subgroup (Table 1, Supplementary Table S1).

For the CRS + HIPEC subgroup (*n* = 13), the study explicitly reported the HIPEC protocol and toxicity profile. HIPEC was delivered with cisplatin 75 mg/m^2^ for 60 min at a target intraperitoneal temperature of 43 °C, using either an open or closed technique according to institutional protocol (Table 1). HIPEC-related toxicity occurred in 2/13 patients (15.3%), consisting of one grade 1–2 acute renal failure and one grade 3 leukopenia. Both adverse events were reversible with conservative management (Table 3).

Perioperative outcomes reported specifically for the CRS + HIPEC subgroup were otherwise limited to these perfusion parameters and HIPEC-related toxicities (Table 3). No perioperative mortality was reported.

At the level of the broader curative-intent cohort (*n* = 20 CRS ± HIPEC), the study reported major postoperative morbidity in approximately 30% of patients and a median postoperative hospital stay of 15 days. However, because these outcomes were not stratified by HIPEC use, they are reported at the cohort level only. From an oncologic standpoint, the curative-intent cohort demonstrated a median overall survival of 61.5 months, measured from the diagnosis of peritoneal metastases, which was markedly longer than the survival observed in patients managed with non-curative approaches (Table 1).

### 3.4. Risk of Bias and Methodological Quality

**Figure 2:**
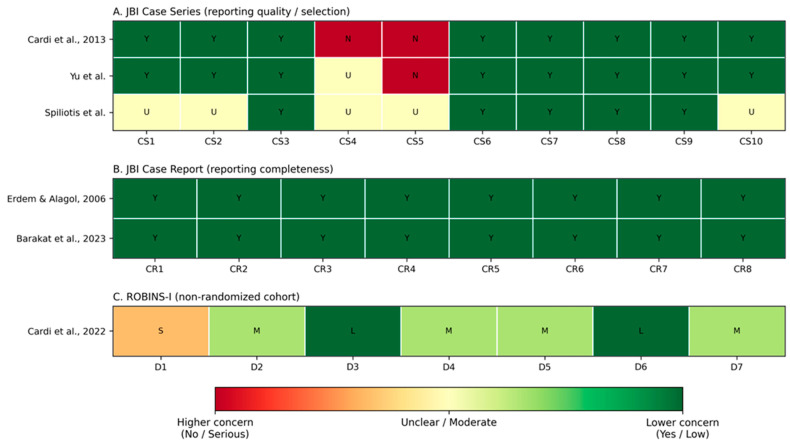
Risk of bias and methodological quality across included studies. Traffic-light plot summarizing domain-level risk-of-bias judgments using design-specific appraisal tools: Joanna Briggs Institute (JBI) critical appraisal checklists for case reports [18,22] and case series [19,20,21], and ROBINS-I (Risk Of Bias In Non-randomized Studies of Interventions) for the multicenter retrospective cohort [23]. Each cell reflects the judgment for the corresponding item/domain within each study; “Yes/Low risk” indicates adequate methodological reporting, whereas “No/Unclear” (JBI) or “Moderate/Serious” (ROBINS-I) indicates increasing concern. CS1–CS10 refer to the 10 items of the JBI critical appraisal checklist for case series. CR1–CR8 refer to the 8 items of the JBI critical appraisal checklist for case reports. D1–D7 refer to ROBINS-I domains.

Risk of bias and methodological quality were assessed using design-specific tools, as summarized in Table 4 and visualized in Figure 2.

Case reports and case series included in the IPD dataset were appraised using the Joanna Briggs Institute (JBI) critical appraisal checklists and were found to have moderate to high risk of selection and publication bias, inherent to their observational and non-consecutive nature. Reporting outcomes and perioperative details were generally adequate, but completeness of inclusion and external validity were limited.

The multicenter cohort study [23], assessed using the ROBINS-I tool, demonstrated serious risk of bias, primarily due to confounding by indication and treatment selection by multidisciplinary teams. The use of HIPEC was not uniform across centers, and outcomes were reported at the cohort level, limiting causal inference regarding the incremental benefit of HIPEC.

A detailed item-level risk-of-bias assessment of study designs is provided in Supplementary Table S2.

## 4. Discussion

### 4.1. Principal Findings

PMBC is an uncommon and clinically challenging manifestation of metastatic breast cancer, associated with poor outcomes in historical palliative series and without a standardized locoregional treatment pathway [3,4,13,24,25]. The present review identified only six peer-reviewed studies reporting CRS plus HIPEC or curative-intent CRS with or without HIPEC in this setting, confirming that the evidence base remains sparse and dominated by case reports, small case series, and one non-randomized multicenter cohort [18,19,20,21,22,23]. The pooled IPD dataset from case reports and case series suggests that CRS plus HIPEC can be delivered with acceptable perioperative morbidity and no reported perioperative mortality in highly selected patients [18,19,20,21,22]. These findings support feasibility, but they should not be interpreted as proof of oncologic efficacy, because the available data are uncontrolled, heterogeneous, and strongly affected by selection and publication bias, as reflected by the design-specific risk-of-bias assessment [16,17].

### 4.2. Clinical Interpretation of Feasibility and Safety

The perioperative findings are clinically relevant because CRS and HIPEC are complex interventions that require advanced peritonectomy techniques, careful patient selection, and experienced multidisciplinary teams [5,6,7,8]. In other peritoneal surface malignancies, the value and safety of CRS with intraperitoneal chemotherapy are closely linked to disease distribution, completeness of cytoreduction, and institutional expertise [9,10,11,26]. In the present case-based IPD dataset, complete cytoreduction (CC-0) was achieved in 9 of 17 patients, major postoperative morbidity occurred in 17.6%, and no perioperative mortality was reported [18,19,20,21,22]. The multicenter cohort similarly reported no perioperative mortality, major morbidity in the broader curative-intent cohort, and reversible HIPEC-related toxicity in the CRS plus HIPEC subgroup [23]. Taken together, these observations suggest that morbidity may be acceptable in specialized centers, but they also emphasize that outcomes are likely center-dependent and cannot be generalized to unselected PMBC patients.

### 4.3. Heterogeneity of Metastatic Phenotype and Treatment Context

A central limitation of the current literature is the heterogeneity of the metastatic phenotype being treated. Included patients differed in synchronous versus metachro-nous PMBC presentation, extent of peritoneal disease, completeness of cytoreduction, presence or control of extra-peritoneal metastases, histologic subtype, receptor profile, and systemic therapy before and after surgery [18,19,20,21,22,23]. This is particularly important in breast cancer, where prognosis and treatment responsiveness are strongly influenced by molecular subtype, hormone-receptor/HER2 status, metastatic burden, and the availability of modern systemic therapy [13,27,28,29]. Population-based data also indicate that peritoneal metastases from extra-abdominal malignancies represent a biologically heterogeneous clinical entity rather than a uniform disease state [24]. In this review, lobular histology was frequent in both the pooled IPD dataset and the multicenter curative-intent cohort, but receptor status and molecular subtype were inconsistently reported in the case-based literature [18,19,20,21,22,23]. These differences confound interpretation because favorable outcomes may reflect tumor biology, indolent disease course, systemic treatment responsiveness, limited metastatic burden, or highly selective surgical referral rather than the independent effect of HIPEC.

### 4.4. Interpretation of the Multicenter Cohort and the Incremental Role of HIPEC

The multicenter cohort by Cardi et al. provides the most informative comparative context because it evaluated curative-intent CRS with or without HIPEC versus non-curative strategies in PMBC [23]. However, this study cannot isolate the incremental benefit of HIPEC. Allocation to curative-intent treatment was non-randomized and based on multidisciplinary assessment, while HIPEC was administered to only a subset of the curative-intent cohort [23]. Baseline variables and oncologic outcomes were not stratified separately for the HIPEC-treated subgroup; therefore, the longer survival reported for the curative-intent cohort should be interpreted primarily as a signal of patient selection and potential value of aggressive multidisciplinary management, rather than evidence that HIPEC itself improves survival [23]. This distinction is essential and is consistent with the serious risk of bias assigned to the cohort using ROBINS-I, particularly with respect to confounding by indication and selection of participants [17].

### 4.5. Clinical Implications and Future Research

Based on the available evidence, CRS plus HIPEC should not be considered a standard treatment for PMBC. Current metastatic breast cancer guidance remains centered on systemic therapy, with locoregional approaches considered only in selected clinical contexts [13,30]. Similarly, consensus guidance on peritoneal surface malignancies recognizes the rarity of breast cancer peritoneal metastases and supports individualized, multidisciplinary decision making rather than routine CRS plus HIPEC [25]. At most, CRS plus HIPEC may represent an investigational strategy for carefully selected patients with limited or controllable peritoneal disease, absence or durable control of extra-peritoneal metastases, favorable biology, and a realistic chance of complete cytoreduction. This cautious interpretation is consistent with the broader literature on oligometastatic breast cancer, in which potential benefit from local therapy appears most plausible in carefully selected patients with low-volume disease and favorable prognostic features [30,31,32,33]. Future prospective multicenter registries should use standardized reporting of PCI, CC score, histology, receptor and molecular subtype, performance status, systemic therapy sequence, extra-peritoneal disease status, HIPEC regimen, complication grading, and time-zero definitions for outcomes. Such data are necessary before comparative studies can clarify whether HIPEC provides incremental benefit beyond CRS and modern systemic therapy.

### 4.6. Strengths and Limitations

This review has several strengths. It used a structured and reproducible search strategy, transparent PRISMA-informed reporting of study selection, design-appropriate risk-of-bias tools, and a deliberately conservative synthesis that separates case-based IPD from the multicenter cohort [14,15,16,17]. The main limitations are those of the underlying literature. The evidence consists of case reports, small case series, and one non-randomized multicenter cohort, with no randomized comparisons, small sample size, selective reporting, and limited external validity [18,19,20,21,22,23]. Important variables such as performance status, systemic therapy timing, PCI, molecular subtype, extra-peritoneal disease, and standardized complication grading were incompletely reported [18,19,20,21,22,23]. There was also substantial heterogeneity in HIPEC agents, perfusion temperature, duration, and technique, as well as inconsistent outcome definitions and follow-up duration [18,19,20,21,22,23]. Consequently, the results should be considered hypothesis-generating only and should not be used to infer the independent oncologic effect of HIPEC.

## 5. Conclusions

Peritoneal metastases from breast cancer remain a rare and clinically challenging pattern of metastatic disease for which no standardized treatment pathway currently exists. The available evidence suggests that cytoreductive surgery combined with HIPEC is technically feasible in a highly selected subset of patients, particularly when peritoneal disease is limited, complete cytoreduction is achievable, extra-peritoneal disease is absent or well controlled, and treatment is delivered within experienced peritoneal surface malignancy centers as part of a multidisciplinary strategy.

However, these findings should be interpreted with caution. The current evidence base is composed mainly of case reports, small case series, and one non-randomized multicenter cohort, with substantial clinical and methodological heterogeneity. Therefore, CRS plus HIPEC should not be regarded as an established standard of care for peritoneal metastases from breast cancer, but rather as a potentially valuable individualized or investigational approach for carefully selected patients.

Future prospective multicenter registries and collaborative comparative studies are needed to define reproducible selection criteria; clarify the prognostic impact of PCI, completeness of cytoreduction, molecular subtype, systemic therapy sequence, and extra-peritoneal disease status; and determine whether HIPEC provides incremental benefit beyond cytoreductive surgery and modern systemic therapy alone.

## Figures and Tables

**Table 1:** Characteristics of included studies and treatment protocols.

Study (Year)	Design	Patients with PMBC (*n*)	Peritoneal-Directed Strategy	HIPEC Regimen (Agent, Dose)	Perfusion Parameters (Time, Temp, Technique)	Outcomes Reported (Time-Zero)	Key Findings	Included in IPD Pooling
Erdem & Alagöl (2006) [18]	Case report	1	CRS + HIPEC	Cisplatin 150 mg in 3 L saline	90 min, 40–42 °C, abdomen closed (closed perfusion circuit)	Follow-up after CRS + HIPEC	No recurrence/metastasis at 40 months. Discharge on POD10. No periop complications reported.	Yes
Cardi et al. (2013) [19]	Case series	5	CRS + HIPEC	Cisplatin 75 mg/m^2^	60 min, 40 °C, closed technique, flow 500 mL/min	Outcomes from CRS + HIPEC	PCI 15–24. CC: CC0 2/5, CC1 2/5, CC2 1/5. OS: 1 death at 56 mo, 4 alive DF at 13/45/74/128 mo	Yes
Yu et al. (2021) [20]	Case series	4	CRS + HIPEC	Docetaxel 120 mg + cisplatin 120 mg (each in 3 L)	60 min total (30 min/drug), 43 ± 0.5 °C, open abdomen-Coliseum	OS from CRS + HIPEC	PCI 21–39. CC: CC0 2/4, CC3 2/4. No periop complications reported. OS 31/28/15/49 mo (all alive at last FU)	Yes
Spiliotis et al. (2021) [21]	Case series	5	CRS + HIPEC	Cisplatin 100 mg/m^2^ + paclitaxel 175 mg/m^2^	60 min, 42.5 °C, closed technique	Survival after CRS + HIPEC	PCI 7–24. CC: CC0 4/5, CC1 1/5. Survival: 78/68/10/54(death)/12 mo	Yes
Barakat et al. (2023) [22]	Case report (2 cases)	2	CRS + HIPEC	Melphalan 50 mg/m^2^	90 min, 41–43 °C, closed technique	OS and PFS from CRS + HIPEC date	PCI exploration 14/29. CC 1 both. ICU 1 day both. LOS 8/13 d. OS 49/38 mo. PFS 5/2 mo. peritoneal recurrence 26 mo in 1 pt	Yes
Cardi et al. (2022) [23]	Multicenter retrospective cohort	49	Curative CRS + HIPEC (*n* = 13), CRS-HIPEC (*n* = 7) Non-curative (*n* = 29)	Cisplatin 75 mg/m^2^	60 min, 43 °C, open/closed per center	OS from PM diagnosis	Curative cohort: PCI median 15 (13–20.5). CC0 13/20. major morbidity 6/20 (30%). HIPEC toxicity 2/13 (15.3%)	No (cohort-level)

Abbreviations: BC: breast cancer, CC: completeness of cytoreduction, CRS: cytoreductive surgery, DF: disease-free, FU: follow-up, HIPEC: hyperthermic intraperitoneal chemotherapy, ICU: intensive care unit, IPD: individual-patient data, LOS: length of stay, OS: overall survival, PCI: peritoneal cancer index, PM: peritoneal metastasis, PMBC: peritoneal metastasis from breast cancer, POD: postoperative day, PFS: progression-free survival.

**Table 2:** Individual-patient baseline and disease characteristics in the pooled IPD dataset (*n* = 17).

Study (Year)	Case	Age at CRS/HIPEC (y)	Interval Time from BC to PM (y)	Histology	ER/PR/HER2 at PM	PCI Score	CC Score
Erdem & Alagöl (2006) [18]	1	55	2	IDC	ER+/PR+/HER2 NR	NR	0
Cardi et al. (2013) [19]	1	58	11	IDC	ER+++/PR−/HER2−	15	0
	2	54	30	ILC	ER+/PR−/HER2++	22	1
	3	55	21	ILC	ER++/PR++/HER2−	22	2
	4	77	14	IDC	ER−/PR−/HER2−	24	1
	5	53	18	IDC	ER++/PR+/HER2−	18	0
Yu et al. (2021) [20]	1	NR	13.3	ILC	ER+/PR+/HER2−	39	3
	2	NR	0	IDC	ER+/PR−/HER2+	28	3
	3	NR	11.2	IDC	ER+/PR−/HER2−	21	0
	4	NR	0	IDC	ER+/PR+/HER2−	30	0
Spiliotis et al. (2021) [21]	1	63	15	IDC	NR	8	0
	2	68	12	ILC	NR	14	0
	3	55	10	ILC	NR	24	1
	4	82	17	IDC	NR	10	0
	5	65	12	ILC	NR	7	0
Barakat et al. (2023) [22]	1	72	NR	ILC	ER+/PR+/HER2−	14	1
	2	59	NR	Ductal-lobular	ER+/PR−/HER2−	29	1

Abbreviations: BC: breast cancer, CC: completeness of cytoreduction, CRS: cytoreductive surgery, ER: estrogen receptor, HER2: human epidermal growth factor receptor 2, HIPEC: hyperthermic intraperitoneal chemotherapy, ILC: invasive lobular carcinoma, IDC: invasive ductal carcinoma, NR: not reported, PCI: peritoneal cancer index, PM: peritoneal metastasis, PR: progesterone receptor.

**Table 3:** Perioperative/postoperative outcomes and survival metrics: IPD dataset vs. multicenter cohort.

Metric	IPD Dataset (*n* = 17)	Multicenter Curative Cohort (CRS ± HIPEC *n* = 20, CRS + HIPEC Subgroup *n* = 13)
HIPEC duration (min)	Mean of 65.3, median of 60 (range of 60–90), *n* = 17	60, *n* = 13
Intraperitoneal temperature (°C)	Range of 40–43	43, *n* = 13
Operative time	Mean of 374.5 min (range of 190–636), *n* = 11	225 min (median, IQR of 200–272.5)
Blood loss	Mean of 944 mL (range of 400–2000), *n* = 9	550 cc (median, IQR of 300–1100)
ICU stay	Mean of 18.9 h (range of 12–24), *n* = 7	12 h (median, IQR of 9–18)
Postop LOS	Mean of 15.1 d (range of 8–24), *n* = 12	15.5 d (median, IQR of 13–20.2)
Major morbidity	3/17 (17.6%) (CTCAE ≥ 3/NCI grade IV where reported)	6/20 (30%) (grade IIIa–IVa)
Perioperative mortality	0	0
HIPEC drug toxicity	Reported sporadically (e.g., transient renal toxicity in Cardi et al. 2013 [19]; hematologic in Barakat et al. 2023 [22])	2/13 (15.3%) (renal failure grade 1–2, leukopenia grade 3, reversible)
OS from CRS/HIPEC	Median not reached, OS at 12 and 36 months of 100%, OS at 60 months of ~53%	OS from PM diagnosis: median of 61.5 months (curative)
Disease control	DFS/PFS variably reported; explicit DF durations of 13–128 mo; PFS of 2–5 mo in Barakat et al. 2023 [22]	Recurrence/progression after CRS: median of 54 months, peritoneal recurrence of 7/20 (35%) after median of 39 months

Abbreviations: CRS: cytoreductive surgery, CTCAE: Common Terminology Criteria for Adverse Events, DFS: disease-free survival, HIPEC: hyperthermic intraperitoneal chemotherapy, ICU: intensive care unit, IQR: interquartile range, LOS: length of stay, NR: not reported, OS: overall survival, PCI: peritoneal cancer index, PFS: progression-free survival, PM: peritoneal metastases.

**Table 4:** Risk of bias and methodological quality assessment.

Study (Year)	Design	Tool Used	Overall Judgment	Main Limitations Driving Bias
Erdem & Alagöl (2006) [18]	Case report	JBI Case Report Checklist	High RoB	Single patient, no comparator, selective reporting, limited generalizability
Cardi et al. (2013) [19]	Case series	JBI Case Series Checklist	Moderate–High RoB	Non-consecutive, small *n*, heterogeneous prior therapy, no control, outcomes reported but limited external validity
Yu et al. (2021) [20]	Case series	JBI Case Series Checklist	Moderate–High RoB	Small *n*, short/heterogeneous follow-up, selection bias, limited adverse-event detail
Spiliotis et al. (2021) [21]	Case series	JBI Case Series Checklist	High RoB	Sparse perioperative reporting, short follow-up for some, potential selection/publication bias
Barakat et al. (2023) [22]	Case report (2 cases)	JBI Case Report Checklist	High RoB	Very small *n*, heavy pre-treatment, outcome heterogeneity, publication bias
Cardi et al. (2022) [23]	Multicenter retrospective cohort	ROBINS-I	Serious RoB	Confounding by indication, non-random HIPEC allocation, baseline/outcomes not stratified for HIPEC subset, heterogeneity across centers

Abbreviations: JBI: Joanna Briggs Institute, ROBINS-I: Risk Of Bias In Non-randomized Studies of Interventions, RoB: risk of bias.

## Data Availability

No new data were created.

## Supplementary Materials

The following supporting information can be downloaded at https://www.mdpi.com/article/10.3390/jcm15124511/s1, Supplementary Section S1: Table S1: Tumor biology details explicitly reported in multicenter cohort, Supplementary Section S2: Table S2: Item-level risk of bias and methodological quality appraisal. Supplementary Section S3: Full search strategy and PRISMA counts.

## Abbreviations

The following abbreviations are used in this manuscript:

BC	Breast cancer
CC	Completeness of cytoreduction (score)
CRS	Cytoreductive surgery
CTCAE	Common Terminology Criteria for Adverse Events
DFS	Disease-free survival
ER	Estrogen receptor
HER2	Human epidermal growth factor receptor 2
HIPEC	Hyperthermic intraperitoneal chemotherapy
ICU	Intensive care unit
IDC	Invasive ductal carcinoma
ILC	Invasive lobular carcinoma
IPD	Individual-patient data
IQR	Interquartile range
JBI	Joanna Briggs Institute
LOS	Length of stay
MDT	Multidisciplinary team
NR	Not reported
OS	Overall survival
PCI	Peritoneal cancer index
PFS	Progression-free survival
PM	Peritoneal metastasis
PMBC	Peritoneal metastasis from breast cancer
POD	Postoperative day
PR	Progesterone receptor
PRISMA	Preferred Reporting Items for Systematic Reviews and Meta-Analyses
RoB	Risk of bias
ROBINS-I	Risk Of Bias In Non-randomized Studies of Interventions
SWiM	Synthesis without meta-analysis
