# Supplementary material for: The Role of Cytoreductive Surgery Plus Hyperthermic Intraperitoneal Chemotherapy (HIPEC) in Peritoneal Metastases from Breast Cancer: A Comprehensive Review and Pooled Individual-Patient Analysis
Source: J Clin Med. 2026 Jun 11;15(12):4511. doi: 10.3390/jcm15124511 (PMC13301833; doi:10.3390/jcm15124511)
Supplement: Supplementary file 1 [file jcm-15-04511-s001.zip › jcm-4328611-supplementary.pdf]

Supplementary Material

Supplementary Section S1: Supplementary Table S1. Tumor biology details explicitly reported in multicenter cohort.

Study	Group CRS ± HIPEC	Histology	ER	PR	HER2	Subtype / Notes
Cardi et al. (2022) [23]	Curative cohort (n=20)	Lobular 13/20; Ductal 7/20	Predominantly ER+	NR	NR	Luminal A 10/20; Luminal B HER2+ 4/20; Luminal B HER2– 4/20; Basal-like 2/20; not stratified for HIPEC-only (n=13).

Abbreviations: ER, estrogen receptor; HER2, human epidermal growth factor receptor 2; NR, not reported; PR, progesterone receptor.

Supplementary Section S2: Supplementary Table S2. Item-level risk of bias and methodological quality appraisal.

S2a. JBI Case Series Checklist (CS1–CS10) judgments.

Study	CS1	CS2	CS3	CS4	CS5	CS6	CS7	CS8	CS9	CS10	Overall concerns
Cardi et al. (2013) [19]	Y	Y	Y	N	N	Y	Y	Y	Y	Y	Moderate–High
Yu et al. (2021) [20]	Y	Y	Y	U	N	Y	Y	Y	Y	Y	Moderate
Spiliotis et al. (2021) [21]	U	U	Y	U	U	Y	Y	Y	Y	U	High

Abbreviations: JBI, Joanna Briggs Institute; Y, yes; N, no; U, unclear/insufficient reporting. CS1–CS10 refer to the 10 items of the JBI Critical Appraisal Checklist for Case Series.

S2b. JBI Case Report Checklist (CR1–CR8) judgments.

Study	CR1	CR2	CR3	CR4	CR5	CR6	CR7	CR8	Overall concerns
Erdem & Alagöl (2006) [18]	Y	Y	Y	Y	Y	Y	Y	Y	Low reporting concerns; inherent selection/publication bias
Barakat et al. (2023) [22]	Y	Y	Y	Y	Y	Y	Y	Y	Low reporting concerns; inherent selection/publication bias

Abbreviations: JBI, Joanna Briggs Institute; Y, yes. CR1–CR8 refer to the 8 items of the JBI Critical Appraisal Checklist for Case Reports.

S2c. ROBINS-I domain judgments (multicenter cohort).

Study	D1 Confounding	D2 Selection	D3 Classification	D4 Deviations	D5 Missing data	D6 Outcomes	D7 Reporting	Overall
Cardi et al. (2022) [23]	Serious	Moderate	Low	Moderate	Moderate	Low	Moderate	Serious

Abbreviations: ROBINS-I, Risk Of Bias In Non-randomized Studies of Interventions; D1–D7, ROBINS-I domains.

Supplementary Section S3. Full search strategy and PRISMA counts.

Last search date: 31 December 2025. Searches were performed in PubMed/MEDLINE, Embase, and Google Scholar. Reference lists of eligible studies were also checked manually. Duplicate records were removed by DOI matching and exact or near-exact title matching, followed by author/year verification where needed.

PubMed/MEDLINE query:

(("breast cancer"[Title/Abstract] OR "breast carcinoma"[Title/Abstract] OR breast[Title/Abstract]) AND (HIPEC[Title/Abstract] OR "hyperthermic intraperitoneal"[Title/Abstract] OR "intraperitoneal hyperthermic"[Title/Abstract] OR IPHC[Title/Abstract]) AND ("peritoneal metastasis"[Title/Abstract] OR "peritoneal metastases"[Title/Abstract] OR "peritoneal carcinomatosis"[Title/Abstract])) AND ("1900/01/01"[Date - Publication] : "2025/12/31"[Date - Publication])

Embase query (Title/Abstract):

('breast cancer':ti,ab OR 'breast carcinoma':ti,ab OR breast:ti,ab) AND ('hyperthermic intraperitoneal chemotherapy':ti,ab OR hipec:ti,ab OR 'intraperitoneal hyperthermic':ti,ab OR iphc:ti,ab) AND ('peritoneal metastasis':ti,ab OR 'peritoneal metastases':ti,ab OR 'peritoneal carcinomatosis':ti,ab) AND [1900-2025]/py

Google Scholar screening:

Google Scholar was queried using equivalent keyword combinations, including: "breast cancer" HIPEC "peritoneal metastases"; "breast carcinoma" "hyperthermic intraperitoneal chemotherapy" "peritoneal carcinomatosis"; and breast IPHC "peritoneal metastasis". The first 1163 relevance-ranked results were screened.

PRISMA counts:

PRISMA item	Count
Records identified (PubMed/MEDLINE)	18
Records identified (Embase)	57
Records screened (Google Scholar)	1163
Total records identified	1238
Duplicates removed	412
Records screened after deduplication	826
Records excluded at title/abstract stage	819
Full-text reports assessed	7
Full-text reports excluded (not peer-reviewed; book chapter)	1
Studies included	6
